# Cyber-Offending and Traditional Offending over the Life-Course: an Empirical Comparison

**DOI:** 10.1007/s40865-018-0087-8

**Published:** 2018-08-01

**Authors:** Marleen Weulen Kranenbarg, Stijn Ruiter, Jean-Louis van Gelder, Wim Bernasco

**Affiliations:** 1 Department of Criminology, Vrije Universiteit Amsterdam, Amsterdam, The Netherlands; 2 Netherlands Institute for the Study of Crime and Law Enforcement, Amsterdam, The Netherlands; 3 Department of Sociology, Utrecht University, Utrecht, The Netherlands; 4 Department of Psychology of Conflict, Risk and Safety, University of Twente, Enschede, The Netherlands; 5 Department of Spatial Economics, Vrije Universiteit Amsterdam, Amsterdam, The Netherlands

**Keywords:** Cybercrime, Traditional crime, Cyber-dependent crime, Life-course, Longitudinal comparison, Fixed effects panel models

## Abstract

**Purpose:**

This paper argues that cyber-dependent offending differs in important ways from other types of offending, which poses challenges to established life-course criminological explanations. Moreover, this study examines to what extent life circumstances in both private and professional life are differentially related to cyber-offending and traditional offending.

**Methods:**

This study analyzes longitudinal registration data of all adults who have been at least once suspected of a cybercrime (*N* = 870) and/or a traditional crime (*N* = 1,144,740) in the Netherlands during the period of 2000–2012. Using fixed effects panel models, within-person effects of household composition, employment, and enrollment in education on the likelihood of cyber-offending are compared with those for traditional offending.

**Results:**

Similar results are found with respect to individual’s private lives. An individual is less likely to commit cybercrime as well as traditional crime in years in which that individual shares a household with a partner, whether with or without children, than in other years. For the professional life, several important differences are found. Employment and enrollment in education are not statistically significantly related to cyber-offending, whereas they reduce the likelihood of traditional offending. In fact, for these professional life circumstances, opposite effects are found in this population.

**Conclusions:**

This first study to empirically compare cyber-offending and traditional offending over the life-course finds important similarities and differences. The results hint at the importance of possible cybercriminal opportunities provided by otherwise preventive professional life circumstances.

## Introduction

The prevalence of traditional crime has been declining for several decades now [54], but cybercrime shows a remarkable upward trend. The rate of computer hacking incidents recorded in Dutch police registration data has tripled between 2007 and 2017 [47, 50]. In 2016, malicious hacking (of computers, email accounts, websites, or online profiles) was the most reported crime in a nationwide representative victimization survey in the Netherlands (4.9% of the population was victimized), followed by vehicle vandalism (4.1%), and bicycle theft (3.8%, [49]).

Given the rise in cybercrimes and the fact that some of their features clearly distinguish them from most traditional crimes, it is important to assess whether established life-course criminological explanations that have been developed for other types of offending are equally informative in understanding cyber-offending over the life-course. For example, there are several reasons why an individual may expect less negative social consequences from committing a cybercrime, compared to committing a traditional crime (e.g., [21, 24, 27, 53, 69]). Similarly, other people may also be less capable of controlling someone’s online behavior compared to the offline behavior. In addition, compared to traditional criminal opportunities, other activities and situations may provide opportunities for committing cybercrime (e.g., [16, 36, 40, 55, 68]). These features make cyber-offending an interesting test case for established life-course criminological findings.

This study examines cyber-dependent crimes [30], i.e., crimes that are “a direct result of computer technology” [14]. These are crimes that cannot be committed without the use of IT-systems (Information Technology) and, therefore, did not exist prior to the advent of those systems. Examples are malicious hacking of computers, email accounts, websites, or online profiles, using malware and blocking access to websites (e.g., by flooding a web server with unwanted traffic, a Distributed Denial of Service (DDoS) attack).^1^ We focus on these cyber-dependent crimes because they provide the sharpest contrast to traditional crimes, as they completely take place in the digital realm. Therefore, these cyber-dependent crimes are expected to differ most from traditional crimes.

This paper presents the first large-scale analysis of cyber-offending over the life-course and examines the extent to which life circumstances in both private and professional life are differentially related to whether an individual commits cybercrime or traditional crime. For an investigation of potential life-course transition effects, it uses unique longitudinal police data and population registration data of all suspects of cybercrime and traditional crime in the Netherlands during the period of 2000–2012. The life circumstances under study are living together with others (e.g., family), being employed, and being enrolled in education. These are life circumstances in which individuals have a higher stake in conformity as they have more to lose when they commit crime (e.g., [17, 43]). Furthermore, in these circumstances, there is more (informal) social control and social support (e.g., [17, 43]) both of which have been found to reduce traditional offending. When individuals live in these circumstances, their daily activities also provide less criminal opportunities than when they would live under different circumstances (e.g., [66]). Although these arguments already have proven merit for explaining traditional offending over the life-course, the question remains whether they can also successfully be applied in the explanation of cyber-offending. After a short summary of theory and research on traditional offending over the life-course, we will discuss arguments that challenge their applicability to cybercrime.

### Offending over the Life-Course

There is an extensive body of criminological literature that shows that some life circumstances reduce the likelihood of offending. Differences in social bonds and social control are suggested to be the main explanations. When individuals have strong relationships with others, they experience both direct and indirect control over their behavior (e.g., [17, 43]). Direct control occurs when significant others disapprove or sanction particular behavior, which is more likely to happen when individuals are in life circumstances during which others are more often around during their daily activities. Indirect control operates through the expectation that sanctioning by others may occur in the future. In order to maintain strong social bonds, individuals invest in their relationships, which increase their stake in conformity. Committing crime jeopardizes these investments. Consequently, the more resources individuals have invested in their relationships, the more they have to lose when they commit a crime, and the less likely they are to do so. In addition, because crimes are often committed during daily activities, life circumstances that provide more criminal opportunities could be important for understanding when individuals are more likely to offend. Many life circumstances provide structured daily activities with few criminal opportunities and often high levels of supervision by others. When individuals are in such circumstances, they are expected to commit less crime than when they lack such structured daily activities and are barely supervised. Besides, in these circumstances, individuals generally also have less time to commit crime than in other circumstances (e.g., [66]). In this study, we focus on life circumstances in both the private as well as the professional life of adults, as both aspects of life influence daily activities and the level of social control individuals experience.

Regarding private life, social control approaches (e.g., [17, 43]) assert that when individuals have invested in a romantic relationship and family life by having children, they have a stronger stake in conformity, which would result in a lower likelihood of offending. Moreover, family life also reduces the time spent in criminogenic settings, which further lowers the opportunity of committing crime [62, 66]. Recent reviews suggest that there is indeed a strong link between marriage and desistance, but cohabitation, union formation, and parenthood seem to have even stronger effects than marital status as such [22, 44]. We therefore focus on household composition and look at whether living together with a romantic partner (whether married or unmarried) and living with a child reduces an individual’s likelihood of committing crime.

With respect to professional life, when individuals have invested in employment, they often commit to that lifestyle and face the risk of losing their job when committing crime. Furthermore, the presence of superiors and co-workers exerts a degree of control over their behavior (e.g., [17, 43]), and employment also structures daily activities and leaves less spare time to spend in criminogenic settings [66] and thus to commit crime (other than workplace crime). Recent reviews indicate that employment indeed reduces the likelihood of offending [22, 23].

Individuals’ educational careers often extend well into adulthood with increased numbers of individuals completing higher education. As a result, they enter the labor market at a later stage than in earlier times [12, 37]. Therefore, the lives of young adults now often include periods during which they still follow some form of education, which makes it important to include enrollment in education in life-course criminological research. After all, if an individual invests in obtaining educational credentials, it increases that individual’s stake in conformity [12, 37]. In the Netherlands, education is only mandatory until the age of 18. Therefore, adults who are still enrolled in education deliberately choose to achieve a certain goal. Similar to employment, enrollment in education makes one spend more time in supervised settings and less time in criminogenic settings [12, 52]. Although research on the effect of school enrollment on offending among adults is virtually non-existent, Stouthamer–Loeber et al. [52] found that both employment and enrollment in education were related to desistance.

In addition to these general patterns in how life circumstances are related to offending, this paper’s focus on cybercrime calls for a discussion of life-course criminological research on specialization and versatility. In general, life-course research has shown that most offenders are generalists, who are quite versatile in the different kinds of crime they commit throughout their life (e.g., [10, 29, 35, 38, 39, 65]). On the other hand, short-term specialization also exists (e.g., [29, 35]). McGloin et al. [29] argued that periods of specialization could be the result of changes in routine activities and increases or decreases in some criminal opportunities related to those activities. For example, employment may reduce opportunities for most types of offending, but it could at the same time increase opportunities for fraud or other white-collar or employment-enabled crimes [39], which may drive an offender to specialize in these types of crime. The life-course specialization literature generally focuses on the question to what extent and when offenders show specialization over their life-course (e.g., [10, 29, 35, 39, 65]) and how this should be measured [13, 35].

Some studies have also focused on specific types of offending and the life circumstances that explain why people may commit those types of crime at specific stages in their life-course. For example, Van Den Berg et al. [58] found that while employment reduces the likelihood of sex-offending, marriage and parenthood did not. In fact, parenthood actually increased the likelihood of child abuse, which is in line with the opportunity arguments of McGloin et al. [29]. Similarly, Mercer et al. [32] found that married men have a higher probability of committing violent offenses, which they hypothesize to be reflecting an increase in domestic violence. With regard to criminal opportunities in professional life, Van Onna et al. [59] show that the stereotypical white-collar offender generally holds a white-collar occupational position, such as a business owner, director, or manager. In the current study, we will make a contribution to the specialization and versatility literature by examining how specific life circumstances are differentially related to cyber-offending and traditional offending.

### Cybercrime

Cyber-dependent crimes provide the sharpest contrast to traditional crimes, as these crimes completely take place in the digital context of IT-systems, whereas the large majority of traditional crimes occur offline. Although computer technology could have been used in the commission of some traditional crimes, those are expected to be a small part of all traditional crimes and the offline counterpart of those crimes already existed before the advent of IT-systems. Those traditional crimes in which IT-systems were used in the commission of the crime are known as cyber-enabled crimes [31], such as online fraud and online harassment. These will be considered traditional crimes in this study, as they cannot be clearly distinguished from offline traditional offenses because they could also be committed without the use of IT-systems, whereas the use of IT-systems is a necessary requirement for the cyber-dependent crimes analyzed in this study. Cyber-dependent crimes are thus expected to differ most and be clearly distinguishable from traditional crimes. Hence, the arguments provided in this section for why life-course transitions would be differently related to cyber-offending and traditional offending are expected to be most applicable to these new cyber-dependent crimes.

Whereas traditional crimes are often studied from a life-course perspective, cyber-offending over the life-course and related private and professional life circumstances have not been studied before. Early applications of criminological theory to cybercrime discuss the digital context in which these crimes take place and the extent to which this challenges important components of traditional criminological theories, mainly focusing on the spatio-temporal components of Routine Activity Theory (e.g., [68]). With respect to empirical work on offending, several cybercrime studies have empirically investigated the applicability of traditional criminological explanations in recent years, mainly focusing on low self-control and social learning (e.g., [19, 20, 28, 33]). These studies mainly focused on juveniles and cyber-enabled crimes, such as online piracy and cyber-bullying. Adults and cyber-dependent crimes received much less attention (for reviews, see [18, 64]).

We identify five arguments which call into question whether the empirical findings found for traditional offending over the life-course are expected to be similar for cyber-offending. First, several authors have argued that people feel as if cyberspace is disconnected from the offline real world [21, 53]. People apparently feel as if their online behavior does not carry any real-world offline consequences. Such a disconnect between people’s offline and online behavior may lead them not to feel responsible for their online actions. Second, because the likelihood of apprehension for cybercrime is extremely low [24, 27, 69], most cyber-offenders never experience any negative social consequences. Consequently, individuals who do have a stake in conformity in the offline world may still commit cybercrimes, as they may not consider the real-world offline consequences of their online criminal behavior. Therefore, strong social bonds like family members may affect cyber-offending less than traditional offending. Third, because online activities tend to be much less conspicuous and more anonymous than most offline behavior, the impact of direct social control and daily activities on cyber-offending may be limited. If so, the mere presence of significant others would exert a lower degree of control over individuals’ online behavior than it does over their offline behavior. In fact, individuals may even be able to commit cybercrime irrespective of whether partners, children, colleagues, employers, teachers, or fellow students are present in the situation. This could be particularly true if the perpetrator has more IT-knowledge than the others, who do not understand what is being done on the IT-system. In addition, as argued by Goldsmith and Brewer [15], the internet enables self-directed learning of criminal IT-skills, in which face-to-face social ties are less important.

Fourth, in relation to the life-course specialization literature discussed above, computers are so widely used in most daily activities, that life circumstances in which individuals normally have less traditional criminal opportunities may provide much more opportunities for cybercrime. For example, when individuals are employed, they use computers more often than when they are not [48]. In addition, having knowledge of and access to a company’s IT-system or its data provides employees with opportunities to commit cybercrimes. Several authors have indeed argued that many cybercrimes against businesses are committed by employees [16, 36, 40]. This makes cybercrimes somewhat similar to many white-collar or employment-enabled crimes, in that, the job actually offers opportunities to commit rather than restraining crime [55]. It stands to reason that employment, especially in the IT-sector, increases an individual’s opportunities for and knowledge about cybercrime. Additionally, some Dutch case studies on organized cybercrime have shown that criminal organizations may directly contact people who work in the IT-sector to help them with the IT-related parts of their crime script [3]. Contrary to what is expected for traditional crime, individuals may therefore actually be more likely to commit cybercrime when they are employed than when they are not, especially when employed in the IT-sector. Similarly, with respect to leisure time, Goldsmith and Brewer [15] argue that the Internet provides a source of leisure activities in which cybercriminal opportunities can easily occur.

Fifth, in addition to being an investment in a certain lifestyle, education can also provide an individual with the knowledge to commit cybercrimes, especially IT-related education. Highly educated people have more IT-knowledge than less educated people [48], which makes them more capable of committing cybercrime. Alongside knowledge, schools and universities also provide their students with access to advanced networked systems without which it is much harder to commit cybercrime [25, 26, 67]. For example, by hacking into a university’s network, an individual can access much greater computer capacity for committing a digital attack than what is possible with only a home computer [7]. Similar to what has been discussed with respect to employment in the IT-sector above, criminal organizations may directly contact IT-students as well [3].

All five arguments above call into question whether findings from life-course criminological research on traditional offending could be replicated for cyber-offending and whether effects will be equally strong. A recent Dutch study disclosed that the age-crime-curves of all suspects of criminal hacking in the Netherlands were rather similar to those of all other Dutch criminal suspects [42]. However, no studies to date have assessed what aspects of an individual’s life are related to whether that individual commits cybercrime and the extent to which the effects of life circumstances are similar or different from the effects found in life-course criminological research on traditional crime [18, 64]. This lack of research is largely due to the limited availability of rich longitudinal data on cyber-offending required for life-course criminological research. For the present study, we collected a unique dataset that allows for the first empirical comparison of cyber-offending and traditional offending over the life-course. With these data, we address the question of whether cyber-offending is related to the same life-course transitions as traditional offending. The main goal of this study is to compare the general patterns for traditional offending over the life-course as described above to those for cyber-offending. In line with the large majority of previous studies, we will therefore not distinguish between different types of traditional offending.

### The Present Study

This study looks at cyber-offending over the life-course to examine the extent to which several aspects of private and professional life are related to whether an individual commits a cybercrime. We combine police data for all suspects of cybercrimes and traditional crimes in the Netherlands for the period of 2000–2012 with population registration data from Statistics Netherlands. These data allow us to estimate fixed effects panel models to obtain the intra-individual effects of changes in household composition, employment, and enrollment in education on cyber-offending and traditional offending. The two models are then compared statistically to examine effect differences. Comparing two models that were both estimated on data from the same source provides the most rigorous test available to date of whether the effects differ between cyber-offending and traditional offending.

In line with theory and previous empirical research, we expect that when an individual cohabits with a partner or child, that individual is less likely to commit a traditional crime than when that individual lives alone. For cybercrime, however, we expect household composition to have no effect. We also expect that employment and enrollment in education decrease the odds of committing traditional crime. However, for cybercrime, we predict the opposite, namely that employment and enrollment education *increase* the odds of committing cybercrime, especially if employed in the IT-sector or enrolled in IT-related education.

## Data and Methods

### Data

This study uses panel data from the years 2000–2012 (with the exception of 2010^2^) on the entire population of adult suspects of crime in the Netherlands (mean age *=* 37.97, *SD =* 13.71; 80% male). The dataset contains information for each year on all variables described below for each individual suspected minimally once of a crime during the period of 2000–2012, aged 18 or older; and registered as a resident of a Dutch municipality (registration is mandatory for all residents in the Netherlands). For the individuals who emigrated or died during the study period, only the years in which they lived in the Netherlands are included in the analysis.

The data used in this study combines the police registration data with the population registration data from Statistics Netherlands. This enables us to analyze differences between cyber-offending and traditional offending over the life-course based on the most comparable large-scale data available today, as the data on both cyber-offending and traditional offending originate from the same sources. Even though the likelihood of apprehension by police may be higher for traditional offenses than for cyber-offenses, there is no reason to expect that this would result in differences in the life circumstances that are related to offending. Therefore, using these data allows for the best possible comparison between cyber-offending and traditional offending over the life-course. The caveats of using this design will be further addressed in the “Conclusion and Discussion” section. Appendix 1 provides more detail about how the dataset was constructed.

For cyber-offending, 870 unique individuals^3^ had been suspected of cybercrime on at least one occasion. For these individuals, 8752 person-years of data were available, which means the dataset contains on average 10.06 (*SD* = 2.90) years per individual. For traditional crimes, 1,144,740 unique individuals had been suspected of a traditional crime at least once. For these individuals, 11,840,665 person-years of data were available, implying an average of 10.34 (*SD* = 2.79) years per individual. As there is a large difference between cyber-offending and traditional offending in the number of person-years that can be analyzed, higher standard errors are expected for the model on cyber-offending.

It appeared 470 individuals had at least once been a suspect of a cybercrime and at least once been suspect of a traditional crime. In 26% of all person-years in which these offenders committed crimes, they committed both a cybercrime and a traditional crime in the same year. Even though we observe this partial overlap at the individual-level, the goal of this study is to show to what extent life circumstances are differentially related to whether an individual commits cybercrime or traditional crime in a particular year. The strength of the intra-individual analyses conducted in this paper is that these individuals could be included in both the analyses of cyber-offending and traditional offending. As the majority of these offenses were not committed in the same year, our analyses show which life circumstances in a specific year are related to cyber-offending or traditional offending in that year. The dependent variable in both models reflects if, in a specific year, an individual was suspected of cybercrime or traditional crime, respectively. So, even though more than half of all cyber-offenders in our sample had also committed a traditional crime in the study period, this versatility at the level of the offender may obscure specialization during particular life circumstances (at the level of the person-year) as suggested in the life-course specialization and versatility literature discussed in the “Introduction” section.

#### Dependent Variables

Data on whether an individual was a suspect of a crime in a particular year were derived from the longitudinal registration system of the Dutch police, which includes every individual for whom a Dutch police department filed a report. Special investigation units that are not part of the police, such as the tax and customs authorities, do not register their suspects in this system. For a more detailed description, see Appendix 1.

Cyber-offending was constructed as a dichotomous variable that indicates whether or not an individual was a suspect of at least one cyber-dependent crime in a given year. The most common cybercrimes in this sample were different forms of system trespassing, ranging from password guessing to advanced hacks.

Traditional offending was also defined as a dichotomous variable that indicates whether or not an individual was a suspect of at least one traditional crime in a given year. The most common traditional crimes in the sample were property crimes (27.89%), violence (21.03%), serious traffic crimes like dangerous driving under the influence alcohol (19.33%), and public order crimes like vandalism (14.99%).

#### Independent Variables

In order to ensure that the private and professional life circumstances (independent variables) described below precede the involvement in cybercrime and traditional crime (dependent variables), all independent variables (unless stated otherwise) reflect an individual’s situation on January 1st of a particular year. For more information on the exact source and construction of the independent variables, see Appendix 1.

For *household composition*, we distinguished between living alone, with a romantic partner (married or unmarried), with a partner and one or more children, with one or more children but without a partner, and in any other type of household. The latter category contains those who lived with their parents (73.60%), lived with others (11.88%), were institutionalized (6.74%), and within a household for which the composition was unknown (7.78%).^4^ In the analyses, “living alone” is used as the reference category.

*Employment* is measured using three dummy variables that indicate whether an individual was not employed, employed outside the IT-sector, or employed in the IT-sector. Employment includes self-employment. For self-employment, there was no information available about an individual’s situation on January 1st; therefore, for self-employment, the employment dummy variable reflects whether an individual was self-employed at any time during a given year. In the analyses, “not employed” is the reference category.

*Education* is also measured using three dummy variables. Because the educational year starts in September, an individual is considered to be enrolled in education on January 1st if that individual started the education in September the year before. We distinguish between not being enrolled in education, being enrolled in non-IT education, and being enrolled in IT-related education. In the analyses, “not in education” is the reference category.

In longitudinal analyses, it is essential to include an *exposure* measure that captures the degree to which an individual was actually at risk of committing a crime that could have been recorded in the police data. We used the number of days in a year that an individual lived in the Netherlands and had not deceased, divided by 365 to obtain a variable that could range from zero to one. This variable does not reflect the situation on January 1st but exposure throughout the entire year. Similarly, although incarceration data were not available, we included as a predictor variable the number of days (also divided by 365) an individual had lived *institutionalized*, because this category includes (but is not restricted to) incarceration.

### Analytical Strategy

Taking advantage of the panel structure of the data, in which repeated measures for the same individuals are available, the hypotheses were tested with fixed effects panel models. These models provide the most rigorous test for intra-individual effects, because they effectively control for all observed and unobserved time-invariant between-individual factors as potential confounds. Additionally, these multivariate models control for the other intra-individual time-varying variables in the model, and therefore, these models provide knowledge on intra-individual changes in life circumstances over time and how these are related to the outcome variable [6].^5^ Since the outcome variables are dichotomous (whether or not an individual was a suspect of crime in a particular year), the fixed effects logit model is most appropriate. The parameter estimates will be presented as odds ratios. The odds ratio for a specific independent variable indicates by which factor the odds of being a suspect change as a function of a one-unit increase in the independent variable.

The standard fixed effects model only controls for time-stable between-person heterogeneity. However, whether individuals become suspects of crime also varies over time due to factors such as the capacity and prioritization of the police. This is especially the case for cybercrime. The knowledge and specialization of the police increased during the study period, which is reflected by a sharp increase in the number of suspects of cybercrime during those years [24]. In addition, the odds that an individual would commit a cybercrime probably also increased due to the increasing availability of IT-systems, computer and social media use, and suitable online targets. Without taking such period effects into account, our results could be biased. We therefore estimate a so-called two-way error component model which controls for age and period effects by including year dummy variables [1].

We use the seemingly unrelated estimation procedure as developed for Stata [63] for testing whether the parameter estimates differ between the cyber-offending and the traditional offending models. This procedure provides a way to statistically test for effect differences between models based on the same, different, or partially overlapping datasets with different sample sizes.

## Results

In this section, we will discuss the fixed effects logit models in which all variables are included simultaneously and in which we also control for age and period effects by including a dummy variable for each year. Multicollinearity was not an issue in these models, as no variance inflation factor was over 1.55. Since we analyze population data and not a mere sample of crime suspects and statistically non-significant effects and differences may reflect important differences within these population data, we do not limit the discussion of our results to statistically significant effects only.

Table 1 shows the estimated odds ratios of the fixed effects logit models for cyber-offending and traditional offending, respectively. The odds ratios represent the change in the odds an individual commits a crime^6^ in a given year with a one unit increase in the independent variable, typically from 0 to 1, holding everything else constant. Odds ratios above one reflect positive effects, and odds ratios below one represent negative effects. For example, Table 1 shows an odds ratio of .69 for living with a partner. This represents a negative effect and means that the odds an individual commits a cybercrime decrease by 31% ((1 − .69) × 100) when an individual changes from living alone to living with a partner (*p* < .05).

**Table 1:** Results of fixed effects models for cyber-offending and traditional offending

Characteristic	Cyber-offending		Traditional offending		Model comparison
	OR	SE	OR	SE	*χ*^2^ (df)
Household composition					11.66 (4)*
Alone	–	–	–		–
With partner	0.69*	0.11	0.79***	0.00	0.75 (1)
With partner and child	0.54***	0.09	0.81***	0.00	5.79 (1)*
With child	1.81*	0.53	1.07***	0.01	2.83 (1)^†^
Other	0.84	0.12	0.98***	0.00	1.04 (1)
Employment					1.40 (2)
Not employed	–	–	–		–
Employed non-IT	0.90	0.10	0.93***	0.00	0.06 (1)
Employed IT	1.14	0.28	0.89***	0.01	1.02 (1)
Education					0.74 (2)
Not in education	–	–	–		–
In non-IT education	1.10	0.24	0.92***	0.01	0.63 (1)
In IT-education	1.06	0.41	0.88***	0.03	0.23 (1)
Exposure days	1.12	0.40	1.39***	0.01	0.38 (1)
Days institutionalized^b^	0.52	0.23	0.69***	0.01	0.34 (1)
All characteristics combined (*χ*^2^(df))					227.74 (21)***
*N* (person-years)	8752		11,840,665		
Unique individuals^a^	870		1,144,740		

†*p* < .10; *p < .05; **p < .01; ***p < .001 (two-tailed)

^a^Absolute numbers of unique suspects are rounded to multiples of ten

^b^Includes (but is not restricted to) incarceration

Separate year dummy variables were included in the models to control for age and period effects, but these are not displayed in the table

*OR* odds ratio

*SE* standard error

*df* degrees of freedom

### Household Composition

In contrast to our expectations, the household composition effects for cyber-offending are in the same direction and even stronger than those for traditional offending. The joint test of effect differences shows a statistically significant difference in household composition effects for cyber-offending and traditional offending (*χ*^2^(4) = 11.66; *p* < .05). For example, while living with a partner and a child decreases the odds an individual commits a cybercrime by 46% (*p* < .001), it decreases the odds of committing a traditional crime by only 19% (*p* < .001). The last column of Table 1 shows that these effects also differ statistically significantly (*χ*^2^(1) = 5.79; *p* < .05). Similarly, living with a partner reduces the odds an individual commits a cybercrime by 31% (*p* < .05), whereas the odds are only reduced by 21% (*p* < .001) for traditional crime. In general, the results show that households with more social control have stronger decreasing effects on cyber-offending than on traditional offending. The results for single-parenthood are, however, unexpected. Compared to the years in which someone lived alone, cyber-offending is much more likely when that person lived in a single-parenthood household (OR, 1.81). The same effect is much smaller for traditional crime (OR, 1.07). Although the effect on cyber-offending appears to be much stronger, the difference in effects is only marginally significant (*χ*^2^(1) = 2.83; *p* < .10).

### Employment

Both models show similar effects for non-IT employment, although the effects for cyber-offending are not statistically significant. Having a job reduces the odds an individual commits a cybercrime or traditional crime by 10 and 7% (*p* < .001), respectively. For IT-employment, however, we find opposite results. It increases the odds of cyber-offending by 14%, whereas it decreases the odds of traditional offending by 11% (*p* < .001). The decreasing effect of IT-employment for traditional offending is statistically significantly stronger than the decreasing effect of general employment for traditional offending (*χ*^2^(1) = 6.36; *p* < .05; results not shown), while IT-employment increases the likelihood of cyber-offending in this population data, although the effect is not statistically significant.

### Education

For enrollment in education, we find opposite effects for cyber-offending and traditional offending. Although the effects are not statistically significant, being enrolled in education increases the odds of committing a cybercrime in this population. Both enrollment in IT-education (OR, .88) and non-IT education (OR, .92) reduces the odds of committing a traditional crime. Although neither the estimates for cyber-offending nor the results of the joint tests of effect differences are statistically significant, the opposite direction of the effect of enrollment in education is an intriguing finding for the population of Dutch crime suspects.

## Conclusion and Discussion

Since cybercrimes possess several unique features not found in most traditional types of crime, they may pose a challenge to established life-course criminological explanations. We examined this issue by investigating cyber-offending over the life-course and comparing the effects of several life-course transitions between cyber-offending and traditional offending. We employed fixed effects logit models on longitudinal population registration data of all adult suspects of cybercrime and traditional crime in the Netherlands from the period of 2000–2012 in order to test whether household composition, employment, and enrollment in education on are differentially related to the likelihood of committing cybercrime or traditional crime. We argued that some life circumstances that have been identified as reducing the likelihood of offending in the life-course literature may not prevent individuals from committing cybercrime, either because they may feel as if their behavior in cyberspace has no real-world consequences or significant others are less capable of controlling individuals’ online behavior. Following that argument, direct and indirect social control exerted by the presence of, for example, family members or co-workers would not reduce cyber-offending as much as it would reduce traditional offending. In line with the life-course specialization and versatility literature, we also suggested that some otherwise preventive life circumstances, like employment, may actually provide more opportunities to commit cybercrime.

Contrary to our expectations, we found that the effects of household composition on cyber-offending were in the same direction and even stronger than those for traditional offending. It appears that when an individual is cohabiting, that individual is less likely to commit a cybercrime than when that individual lives alone. Although we argued that exerting social control on individuals’ online behavior is difficult, these results seem to suggest that family members may still have an inhibiting effect, which reduces the likelihood of cyber-offending. Possibly, it is a misguided assumption that spending more time at home with a family would expose an individual to relatively more opportunities for committing cybercrime than traditional crime. Spending time at home with family may, for example, reduce the time an individual can spend alone behind a computer. Another possible explanation is that the effect of opportunities in private life is offset by the indirect social control exerted by family members. These results seem to suggest that *indirect* social control exerted by strong social bonds, such as family members, may be able to reduce the likelihood of offending, even for crimes that are committed in such a way that exerting *direct* social control seems less possible, and where the chances of getting caught are relatively low. However, the results also showed that, compared to living alone, single-parenthood increased an individual’s likelihood of committing a cybercrime and somewhat increased the likelihood of that individual committing a traditional crime. The partner in a household seems to be the most important source of informal social control, especially for cyber-offending. Future research should examine possible explanations for this larger positive effect for cybercrime. These possible explanations could, for example, be that single-parenthood increases opportunities for cybercrime, or that it decreases direct or indirect social control over online behavior.

In line with our expectations, we did not find a strong and statistically significant protective effect of employment and enrollment in education on cyber-offending. In the complete offender population data used in this study, we even found that employment in the IT-sector and enrollment in education increase the odds that an individual commits a cybercrime, while these circumstances decrease the odds of committing a traditional crime. However, non-IT employment decreases the odds of both cyber-offending and traditional offending. This may suggest that professional life circumstances like employment, in which there generally is more social control and a reduction in criminal opportunities in daily activities, could prevent an individual from committing a cybercrime in general. On the other hand, some traditionally non-criminogenic settings such as IT-employment and education may provide opportunities to commit cybercrimes specifically, which is in line with what is suggested in the life-course specialization literature. Therefore, the results seem to suggest that, in employment settings, an increase or reduction in opportunities for cyber-offending may be more important than an increase or reduction in social control from, for example, colleagues. It should be stressed, however, that the latter results were not statistically significant for cyber-offending, but nevertheless are important given the fact that we analyze full population data. Future research should therefore examine if results can be replicated in different study areas and different time periods. It also seems important to identify the micro-situations in individuals’ daily lives that expose them to opportunities for committing cybercrime.

Based on the results discussed above, some contributions to the life-course literature need to be highlighted. We utilized the unique nature of new crimes, cyber-dependent crimes, to look at established life-course predictors for offending and desistance. In this way, we examined if these traditional life-course explanations for offending also provide insight into offending in a new and different context, where exerting direct social control is argued to be much harder and where offending may require different opportunities and skills. The results suggest that, for these new crimes, indirect social control of strong social bonds [17, 43], especially in an individual’s private life, could be more important than direct social control of these social bonds. This could be derived from the fact that we examined cyber-offending for which direct social control is much harder, while cohabiting with a family is still an important predictor of this cyber-offending. Subsequently, in professional life, opportunities for cyber-offending seem to be more important than direct or indirect social control by others [8, 66]. This is based on the results for this offender population that suggest that only if employment clearly increases opportunities for cyber-offending (i.e., employment in the IT-sector), this is related to an increase in the odds for cyber-offending. As there is no clear reason to believe that in other types of employment there is more direct or indirect social control (especially as IT-employment had a stronger reducing effect on traditional offending than non-IT employment), this difference is expected to be the result of a difference in opportunities for cyber-offending between employment in or outside the IT-sector. These possible explanations for our findings could be further examined in future research on cyber-offending as well as traditional offending over the life-course. These studies should differentiate between the effect of direct social control, indirect social control, and opportunities in the private and professional life.

Several limitations of this study also require discussion. Fixed effects panel models provide a rigorous way for testing effects of life-course transitions because they eliminate all time-invariant (observed and unobserved) between-individual heterogeneity that could possibly confound the effects of interest. Therefore, these models better justify causal claims than most other methods for analyzing observational panel data. However, fixed effects panel models cannot account for unmeasured time-varying factors that may have influenced the likelihood of offending. For example, individuals become involved in romantic relationships without living together or change their daily activities for reasons unrelated to family life, employment, or education. We have no way of knowing whether such changes in individuals’ lives biased our results. However, we did include several indicators for both private and professional lives of individuals that were identified to be most important in the traditional life-course criminological literature. Instead of studying marital status and parenthood, we analyzed the effect of an individual’s household composition, which better captures the actual situation an individual lives in. We took care to ensure that the causal order of the variables was correct by using the situation on January 1st to construct most of our independent variables. However, because the crime data were only available at the annual level, we cannot be sure an individual was still in that same situation at the time of the offense.

Another important point for discussion is that this study had to rely on police suspect data as no self-report or conviction data were available. This means that it is unknown to what extent an individual also committed crimes in the years he or she was not registered as a suspect. In addition, whether the suspects were actually guilty of committing the crimes for which they were a suspect is unclear. However, about 90% of all suspects in the police registration system are eventually convicted in a criminal court or their cases get settled out-of-court by the public prosecutor [5]. It is also difficult if not impossible to generalize our results to the cyber-offender population because so many cyber-offenders operate from other countries and many do not come into contact with Dutch police. It has for example been argued that the most technically skilled cyber-offenders operate from other countries [11]. In addition, in Dutch police records as used in this study, cybercrimes that require advanced technical knowledge cannot be distinguished from those that do not [24, 51]. This lack of specificity in the outcome variable means that cybercrimes that require advanced technical knowledge were combined with cases in which the suspect, for example, only guessed another individual’s password to break into a computer system. Should such distinction have been possible, it would have been interesting to test whether enrollment in IT-education and IT-employment more strongly related to the technically complex forms of cyber-offending. Future research should further investigate the knowledge and opportunities needed for more technically complex cybercrimes and the extent to which these are related to specific life circumstances, specifically in the professional life, especially given our findings that suggest opportunities in professional life seem to be the most important difference between traditional offending and cyber-offending.

The advantage of using police registration data is that they provide information on all suspects of crime instead of a sample. Even parameter estimates that were not statistically significant still reflect how offending is related to important life-course transitions among all suspect of crime in the entire Netherlands in a 13-year period. These data currently provide the best way to rigorously compare cyber-offending with traditional offending because the data for both types of offending originated from the same source and potentially more representative data such as longitudinal self-report data including both cyber-offending and traditional offending have not yet become available, and the data used in this study are more comparable than any data that would be derived from two different sources. Nevertheless, the results should be interpreted with some caution, as research indicates that apprehension rates for cybercrime are very low [24] and probably much lower than for traditional crime. There are several reasons why this may be the case, for example, victims are less likely to report cyber-victimization to the police than traditional victimization, and the police lack the capacity and technical expertise to investigate a cybercrime and identify a suspect [24]. This may have resulted in a more selective sample for cyber-offending compared to traditional offending, and this could have reduced the number of years in which an individual was registered as a cybercrime suspect. Thus, even though the data originate from the same source, the comparability of the data for both types of offending may be somewhat limited. Nevertheless, as our analyses include fixed effects for individuals and years the estimated effects thus best reflect the intra-individual differences over the life-course and any initial differences between individuals do not affect our findings. Besides, we have no reason to expect that the differences in apprehension rates are related to the life circumstances under study.

In our analyses, we compared a specific type of crime, cybercrime, with a diverse set of traditional crimes. As this is the first study that compares cyber-offending and traditional offending over the life-course, this comparison of general patterns in the life-course addressed the most important research question at this moment. Future research may disaggregate the dependent variable and test whether stronger similarities are found if cyber-offending is compared with specific types of traditional offending, for example employment-enabled crimes ([39, 59]). In such studies, the effect of IT-employment should then be compared to the effect of specific types of employment that enable white-collar crimes. To illustrate, future studies could address the question whether the effect of following education in finance or employment in the finance sector on committing fraud is similar to the effect of following IT-education or IT-employment on cyber-offending. This could further advance the literature on specialization over the life-course and enhance our knowledge of the extent to which increases in daily opportunities in the professional life are related to specific types of offending.

In addition, future research with more in-depth data about the offenses may be able to distinguish between traditional offenses that completely take place offline and cyber-enabled traditional offenses that are partly committed online. In that research, it would be possible to see to what extent the differences that are found in this study are also found in a comparison between offline traditional offenses and cyber-enabled traditional offenses. Subsequently, with that type of data, it will also be possible to distinguish between criminal cases in which all criminal acts are completely committed online or offline and cases in which the offender combines offline offenses, cyber-enabled offenses, and cyber-dependent offenses. Similarly, data on co-offending could inform us about the extent to which life circumstances provide opportunities to meet co-offenders. For cybercrime, both online and offline co-offenders could be important, and future life-course research on co-offending, in relation to life circumstances and daily online and offline activities, will provide important insights in the opportunities for meeting online and offline co-offenders in certain life circumstances.

Compared to the large and strong body of life-course research on traditional crimes, our research based on registration data obviously had some limitations, but it provides unique insights into differences and similarities between cyber-offending and traditional offending over the life-course, and it should spark research initiatives that address how these similarities and differences are related to social bonds, social control, and routine activities and opportunities. By using fixed effects panel models to study cyber-offending and traditional offending simultaneously, we generated results that are new to cybercrime and life-course research. To further advance the field, new life-course research is needed to replicate these findings among different populations. Longitudinal self-report life-course studies are advised to start including questions on cyber-offending because that could further enhance our knowledge of when in life individuals are more likely to engage in cybercrime. Such studies could also include detailed questions on the strengths of social bonds and individuals’ actual daily activities and related opportunities, since these cannot be measured in studies that use registration data. For example, these studies could address whether the effect differences of non-IT employment and IT-employment on cyber-offending are the result of differences in opportunities, social bonds and social control, job quality, or financial characteristics of these jobs. As previous research on traditional crime has shown (e.g., [9, 56, 60]), several different aspects of employment could be the reason for its crime-reducing effects. To date, no detailed longitudinal data on cyber-offending and different aspects of important life circumstances are available. Therefore, the field would greatly benefit from studies that look at specific aspects of life circumstances that are related to an increase or decrease in cyber-offending. Specifically, more knowledge is needed about the way IT-employment and education could provide opportunities for cybercrime, as this could guide us to prevention methods for cyber-offending.

With this paper, we demonstrated the usefulness of studying cyber-offending over the life-course. We examined whether established life-course criminological findings for traditional offending also provide insight into cyber-offending, a new type of crime that takes place in a new and different context. The comparison shows similar results with respect to individuals’ private lives, but the results also stress the importance of considering the possible cybercriminal opportunities provided by otherwise preventive professional life circumstances, in particular IT-related employment and enrollment in education.

## Appendix 1: dataset composition

The dataset was constructed by using several individual-level datasets provided by Statistics Netherlands. To facilitate replication, a list of names, in Dutch, of all the datasets used is provided at the end of this Appendix. The individual-level datasets were anonymized and included a non-informative unique personal identification number. We combined the data using these unique identifiers. Below, we describe each dataset in more detail.

### Dependent variables

Data on crime suspects were derived from the police registration system *Herkenningsdienstsysteem*, a longitudinal registration system of the Dutch police that includes every individual for whom a police department filed a report. Special investigation units that are not part of the police, such as tax and customs authorities, do not register their suspects in this system. This means that some economic crimes, environmental offenses, or benefit frauds are not registered in this system. For a more detailed description, see Bernasco [2].

In the Netherlands, the cybercrimes that have emerged as “a direct result of computer technology” [14] are criminalized under specific articles of Dutch criminal law [34], which were used to determine whether a crime was a cybercrime or a traditional crime.

### Independent variables

Several individual-level datasets were based on the Dutch registration system of municipalities, the *Basisregistratie personen* (Dutch acronym: BRP). For more information about this nationwide system, see Blokland and Nieuwbeerta [4]. For our analyses, we extracted date of birth, gender, ethnicity, days living in the Netherlands, days alive, and household composition from the Statistics Netherlands’ individual-level datasets on demographics, international immigration, deceased individuals, and households of all individuals who are registered in BRP. The dataset on households is almost completely derived from the BRP. Only 5 % of the information on household compositions is based on registers of taxes, income support, governmental funding on healthcare, and rental allowance. Another 5 % is imputed by using information from the Labour Force Survey (in Dutch: Enquête Beroepsbevolking; for more information see: [45]).

Employment and self-employment were derived from individual-level datasets on job characteristics, yearly job summary statistics, business characteristics, and individuals who had taxable income out of their own business. These datasets are a combination of data from registration of income taxes, administration of employee insurance, the Survey on Employment and Wages, the Earnings Production System, and the registration system of self-employment. Employment in the IT-sector was constructed using the SBI classification system, which is based on the NACE of the European Union and the ISIC of the United Nations. For the years 2000–2005, we used the SBI 1993 classification (classification numbers 7210, 7221, 7222, 7230, 7260) and, for the years 2006–2016, we used the SBI 2008 classification (classification numbers 6201, 6202, 6203, 6209) to identify IT-employment. These classification numbers include the following sectors: developing and producing software, hardware consultancy, software consultancy, computer facilities management, and software implementation. For more information, see Statistics Netherlands [46].

Whether or not an individual was enrolled in education was derived from the individual-level dataset on highest education. We used changes in completed educational level and attended educational level to derive the start and end dates of a specific education. An individual was considered to be enrolled in education between the years in which he or she started and ended the education. In addition, if an individual started education that in general takes more years than the remaining years in the dataset, the individual was considered to be enrolled in education from the start until the last year included in the dataset. This could have caused a slight overestimation of the number of individuals enrolled in education, as it was not possible to exclude those who had terminated their study. In a similar way, individuals who completed an education that generally takes more years than covered by the dataset were also considered to be enrolled in education until the moment they ended that education. As this variable is constructed by using changes within the period of 2000–2012 in an individual’s formally registered educational level and qualifications, it does not reflect non-registered education and it may slightly underestimate the number of individuals enrolled in education, since it cannot detect individuals who are enrolled in education but do not change their educational level during the period of 2000–2012. This dataset is a combination of data from registers for government-funded high schools, secondary vocational education and adult education, the Central Register of Higher Education Programs, the exam register for secondary education, registration of governmental student financing, the governmental employee insurance agency, and the Labour Force Survey. IT-education was constructed using the International Standard Classification of Education ISCED 1997 [57]. For identifying IT-education, we used the category “computing” (field of education number 48), which are computer sciences or education like: system design, computer programming, data processing, networks, operating systems, and software development.

Combining all these separate datasets resulted in a person-year dataset. Each observation in the dataset contained information on all variables for one specific year for one individual. The used micro datasets are named:

- BAANKENMERKENBUS
- BEBUS
- GBAHUISHOUDENSBUS
- GBAMIGRATIEBUS
- GBAOVERLIJDENTAB
- GBAPERSOONTAB
- HKS (land_delikt & land_ant_del)
- HOOGSTEOPLTAB
- ZELFSTANDIGENTAB

For more information about the used datasets see https://www.cbs.nl/en-gb/our-services/customised-services-microdata/microdata-conducting-your-own-research

## Footnotes

1 These crimes have also been referred to as cyber-trespass by [61]. Although it may seem that committing these crimes requires advanced technical skills, it may actually require little technical expertise. Hacking an email account, for example, can be done in a technically advanced way, but also by just guessing a password. In Dutch police records, it is unfortunately impossible to systematically distinguish cybercrimes that require advanced technical knowledge and skills from those that do not [24, 51].

2 On October 1st, 2010, the Dutch criminal law on malicious hacking changed. Previously, unauthorized access into an IT-system was criminalized under criminal law 138a. From the date of implementation, squatting a house was criminalized by 138a. Because the data are only available at the annual level, it is impossible to distinguish the individuals who were suspected of malicious hacking from those suspected of squatting in 2010. We therefore excluded the year 2010 from the analysis as presented here. However, as a robustness check, we also estimated our models 10 times using all data from 2000 to 2012 while randomly assigning a weighted proportion of the 138a suspects to the individuals who committed a cybercrime in 2010 and subsequently applying Rubin’s formulae [41] to calculate the overall effect sizes and standard errors. The results were virtually identical to those presented here and are available upon request from the first author.

3 Statistics Netherlands requires rounding of absolute numbers about suspects of crime to multiples of 10 and percentages to natural numbers.

4 99.86% of the unknown category immigrated to the Netherlands during the given year, and therefore, the household composition on January 1st was unknown. We also estimated models with dummy variables for all household compositions separately, but all other estimates in the models were largely the same. The results of the additional models are available upon request from the first author.

5 Looking at intra-individual differences implies that some intra-individual variation must exist in the dependent variable. Fixed effects models, therefore, require that every individual in the analysis had at least 1 year of offending and at least 1 year of non-offending. For this reason, the 9180 individuals of whom only information on a single year was known, and the 7460 individuals who were a suspect of crime in every year during the study period were excluded from the analysis.

6 It should be stressed that the outcome variables actually represent being registered as a cybercrime or traditional suspect in the police registration data. It is unknown to what extent an individual also committed crimes in the years he or she was not registered as a suspect. We discuss possible differences in the likelihood of getting registered as a suspect of a cybercrime compared to a registration for traditional crime in the “Conclusion and Discussion” section.
